# Experience of, awareness of and help-seeking for potential cancer symptoms in smokers and non-smokers: A cross-sectional study

**DOI:** 10.1371/journal.pone.0183647

**Published:** 2017-08-28

**Authors:** Julie Walabyeki, Joy Adamson, Hannah L. Buckley, Helena Sinclair, Karl Atkin, Hilary Graham, Katriina Whitaker, Jane Wardle, Una Macleod

**Affiliations:** 1 Hull York Medical School, University of Hull, Hull, United Kingdom; 2 Institute of Health & Society, Newcastle University, Newcastle upon Tyne, United Kingdom; 3 Department of Health Sciences, University of York, Heslington, York, United Kingdom; 4 School of Health Sciences, University of Surrey, Guildford, Surrey, United Kingdom; 5 Health Behaviour Research Centre, University College London, London, United Kingdom; H. Lee Moffitt Cancer Center, UNITED STATES

## Abstract

**Background:**

Presenting to primary care with potential cancer symptoms is contingent on one’s ability to recognize potentially serious symptoms. We investigated differences between smokers and non-smokers in symptoms experienced, awareness and consulting of potential respiratory, head and neck cancer symptoms.

**Methods:**

Smokers and non-smokers aged over 50 from Yorkshire general practice lists were sent a postal questionnaire asking about symptoms, consulting and awareness of cancer symptoms. Data were analysed using STATA14.

**Results:**

Response rate after one reminder was 30.5% (1205/3954). Smoking status was associated with experience of cough (p<0.001), breathlessness (p = 0.002) and tiredness (p = 0.004) with smokers (25.8% of population) more likely than never-smokers (53.6% of population) to experience all three symptoms (cough OR = 2.56;95%CI[1.75–3.75], breathlessness OR = 2.39;95%CI[1.43–4.00], tiredness OR = 1.57;95%CI[1.12–2.19]). Smoking status was associated with awareness of breathlessness as a potential cancer symptom (p = 0.035) and consulting for cough (p = 0.011) with smokers less likely to consult than never-smokers (OR = 0.37;95% CI[0.17–0.80]).

**Conclusion:**

Our findings suggest that current smokers are more likely to experience cough, breathlessness and tiredness, but are less likely to consult for cough than never-smokers. To increase cancer awareness and promote consulting among smokers, innovative interventions improving symptom recognition and empowering smokers to seek help are required.

## Introduction

Cancer remains one of the UK’s major cause of ill-health, both in terms of morbidity and mortality [1]. There has been increasing interest in how patients and professionals recognise cancer symptoms [2] particularly since data show that one-year survival figures for many cancers are poorer in the UK than in comparable European countries [3]. This suggests that people in the UK are diagnosed at a later point in their cancer history than others in Europe, leading to the question of why there should be this apparent delay in diagnosis [4].

Recognition of potentially serious symptoms by patients precedes presentation to primary care and is a key aspect of the pathway to cancer diagnosis [5, 6]. Much of the research has focused on symptom awareness and indicates that not only is there relatively low awareness of cancer symptoms in UK [7, 8], but also higher perceived barriers to presentation including normalization of symptoms [9]; interpretation of symptoms based on previous experiences [10], fear [11], worrying about wasting the doctor’s time [12] and living alone [13].

In seeking to impact on presentation to primary care with potential cancer symptoms, it is worthwhile to consider the groups most at risk of developing cancer in order to understand their awareness of symptoms and how they respond. For example, smoking is associated with increased risk of several cancers, including head and neck [14, 15] (relative risk [RR] larynx = 6.14,95%CI [4.55–8.30]; RR oesophageal = 2.14, 95% CI [1.73–2.65]; RR oropharyngeal = 2.30, 95%CI [1.94–2.72] and lung cancers (RR lung = 9.28 (95%CI [8.31–10.4]) [16] which, if diagnosed early, can be treated more successfully with improved outcomes. However, there is low awareness of potential lung cancer symptoms among smokers [17] although evidence suggests that there is no significant difference in symptom recognition among current or former smokers [18]. Long–term smokers have been shown to be more likely to present later to primary care with potential lung cancer symptoms [13, 19]. However, there is currently limited knowledge of symptom awareness and primary care consultation by smoking status for head and neck cancers.

Through the identification of those groups at higher risk of cancer, but who are also more likely to have lower awareness of and less likely to consult with symptoms, we can target campaigns that have been shown to be effective. For example, public health campaigns have been associated with improving symptom awareness following the first lung cancer campaign in England. The ‘spontaneous awareness’ (when respondents were asked to name as many symptoms of lung cancer as possible) of cough as a lung cancer symptom increased from 54% to 65% post-campaign (p<0.001) [20]. ‘Prompted awareness’ (when respondents were shown a list of lung cancer symptoms and asked whether they could be a warning sign of lung cancer) rose to 33% post-campaign from 18% pre-campaign (p<0.001) [20]. An increased number of lung cancer diagnoses were also observed, alongside a shift in stage distribution and surgical resections [21].

We therefore sought to investigate the symptom experience, awareness of, and primary care consultation for symptoms associated with lung and head and neck cancer among people aged over fifty years in Hull and Leeds according to smoking status.

## Materials and methods

### Setting and governance

We conducted a cross-sectional questionnaire study between October 2013 and March 2014 in Hull and Leeds in the Yorkshire and the Humber region (YH). Hull had the highest smoking prevalence (26.4%) in YH (YH prevalence = 20.1%) in 2014, which was also much higher than the national prevalence (18.0%) according to the Integrated Household Survey (IHS) [22]. The prevalence in Leeds was also higher than national prevalence and the fourth highest in YH (23.1%). Ethical approval was received from the NHS YH-Leeds East Research Ethics Committee (Reference: 12/YH/0341).

### Participants

#### Sample size estimation

We selected cough as our primary symptom as this was likely to be the commonest symptom that could potentially be associated with the cancers of interest [23]. A community survey conducted in Leeds and Bradford estimated the chronic cough prevalence in smokers and non-smokers as 18% and 11% respectively [24]. In order to detect a difference of this magnitude, with 90% power, a sample size of 558 smokers and 558 never-smokers was required. We estimated a response rate of 35% (a conservative estimate of overall response) [25].

### Data collection

The questionnaire development was informed by the: Model of Pathways to Treatment [26], Cancer Awareness Measure (CAM) [27] and Symptoms Appraisal questionnaire [25]. The questionnaire comprised sections on the experience of symptoms of lung and head and neck cancers, pre-existing illnesses/conditions, response to symptoms, triggers to consultation, understanding/awareness of potential cancer symptoms, symptoms interpretation, health services utilization and socio-demographic data (S1 Questionnaire). ‘Cancer’ was not mentioned in the participant information sheet (PIS) or questionnaire. It was only mentioned in the section that explored the respondents’ understanding of potential cancer symptoms, which included cancer as one of the four illnesses (others were ‘flu’, heart disease and asthma). After piloting, patients over fifty years (smokers and practice-matched non-smokers) from GP lists in Hull and Leeds were sent a postal questionnaire (with one reminder). We regarded receiving a completed questionnaire from a respondent as consent to participate in the study.

#### Piloting

We recruited 34 volunteers over 50 years old to pilot the questionnaire, 11 were University of Hull staff members, 4 were members of the public in Hull and 20 members of the public in Nottingham. Most volunteers were White British (88.2%) and female (67.6%). The volunteers completed the questionnaire in the presence of the researcher (JuW). On completion, the respondent was asked to comment on the readability, structure and feasibility of the questionnaire. A number of issues were raised; for example, a number of respondents asked what the term ‘persistent’ meant in the ‘Experience of symptoms’ section (pages 6–9, S1 Questionnaire). This was changed to ‘does/did not go away’ by the researchers (S1 Questionnaire)

#### Recruitment

Eight practices identified by the National Institute for Health Research Clinical Research Network (NIHR CRN) were recruited. Each practice was asked to recruit 500 eligible participants, stratified by smoking status and gender. The eligible individuals were identified using a database search. A GP then screened the list for any patients: who were unable to give informed consent, with a lung or head and neck cancer diagnosis or unsuitable for home visits. The practices identified 3954 eligible patients who were sent packs (comprising the questionnaire, GP letter to patient, a complimentary slip, PIS, and a reply-paid envelope) via post. A secure, confidential electronic file linking a questionnaire unique identifier with each patient was created and stored by each practice. About three months later, reminders were sent once to non-respondents. The practices also provided anonymized, general demographic information (age and gender) for 2707 (68.5%) non-respondents. We checked for the study population representativeness using age and gender.

### Measurements

#### Explanatory variables

Smoking status: Consistent with definitions used within NHS England [28], respondents were asked whether they had ever smoked and those who had not were regarded as never-smokers. Ex-smokers were those reporting that they had previously but did not currently smoke. Smokers were those who reported that they were current smokers.

Respondents were asked to report on their smoking habits; this included the type (cigarettes, including hand-rolled, cigars and pipes) and amount smoked. We used the Centers for Disease Control and Prevention (CDC) conversion to convert the tobacco and cigars to the cigarette equivalent [29]: 0.0325 ounces (oz) = 0.9g = 1 cigarette; we modified this to 1 oz = 31 cigarettes; 1 cigar = 20 cigarettes. We graded the number of cigarettes smoked per day according to the Office for National Statistics (ONS) [30], that is, light smokers = 0–9 cigarettes/day; moderate smokers = 10–19 cigarettes/day and heavy smokers = 20+ cigarettes/day. The very low rate smokers (those who smoke less than one cigarette per day) were also included in the light smokers’ category [31].

#### Outcome variables

Symptoms experience (reported symptoms): Respondents were asked whether they had experienced any of seventeen symptoms (yes/no) in three months prior completing the questionnaire. The symptoms were persistent cough, persistent shoulder pain, unexplained breathlessness, tiredness, unexplained weight loss, persistent chest infection, persistent chest pain, coughing up blood, painful cough, loss of appetite, difficulty swallowing, change in existing cough, persistent mouth ulcer, numbness of lip/tongue, persistent cold sore/cut on lip, hoarse voice for more than 3 weeks and neck lump.

Self-reported consulting for at least one potential cancer symptom (consulting): Respondents were asked to indicate whether or not they had seen the doctor for the experienced symptom listed above (yes/no).

Awareness of at least one potential cancer symptom (awareness): The respondents’ understanding of illnesses was sought by asking them to match fifteen symptoms to four illnesses (including cancer). The fifteen symptoms were persistent cough, persistent shoulder pain, unexplained breathlessness, tiredness, unexplained weight loss, persistent chest pain, coughing up blood, difficulty swallowing, persistent mouth ulcer, persistent cold sore/cut on lip, hoarse voice for more than 3 weeks, neck lump, unexplained pain, sore throat and ‘feeling your heart pound or race’. We excluded ‘feeling your heart pound or race’ in the ‘Awareness’ section from our analysis because it is not a potential lung or head and neck cancer symptom and is associated more with heart disease. If the respondent identified a symptom as a potential cancer symptom, they were considered to be aware of that symptom (yes/no). Please note that some of the symptoms in the ‘Symptoms experienced’ section were not in the ‘Awareness’ section and vice versa.

### Analysis

Baseline characteristics were produced for broad demographic indicators, smoking status, ‘having cancer experience’ and ‘having a previous cardiorespiratory diagnosis’. We examined differences in the socio-demographic characteristics by smoking status. The proportions of symptoms experienced, self-reported consulting and awareness of potential cancer symptoms were also examined. The strength of association between smoking status and: symptoms experienced; self-reported consulting; and awareness, for cough, breathlessness, tiredness and shoulder pain was assessed (Chi-squared test). Logistic regression was used to investigate the relationship between smoking status and awareness of, experience of and self-reported consultation for cough, breathlessness, tiredness and shoulder pain. Adjustment for demographic characteristics (age, gender, education and accommodation), previous cancer experience and cardiorespiratory diagnosis was made in the models. Analyses focused on these symptoms as they were most commonly reported by our study population and in previous research [32]. Cough, breathlessness and tiredness are among the six key symptoms included in the National Institute for Health and Care Excellence (NICE) guidelines for suspected lung cancer [33]. Additionally, cough and breathlessness are part of the national cancer awareness campaigns in England [34]. We regarded missing data as missing-completely-at-random (MCAR) [35]. Data were analyzed using STATA 14.

## Results

**Fig 1:**
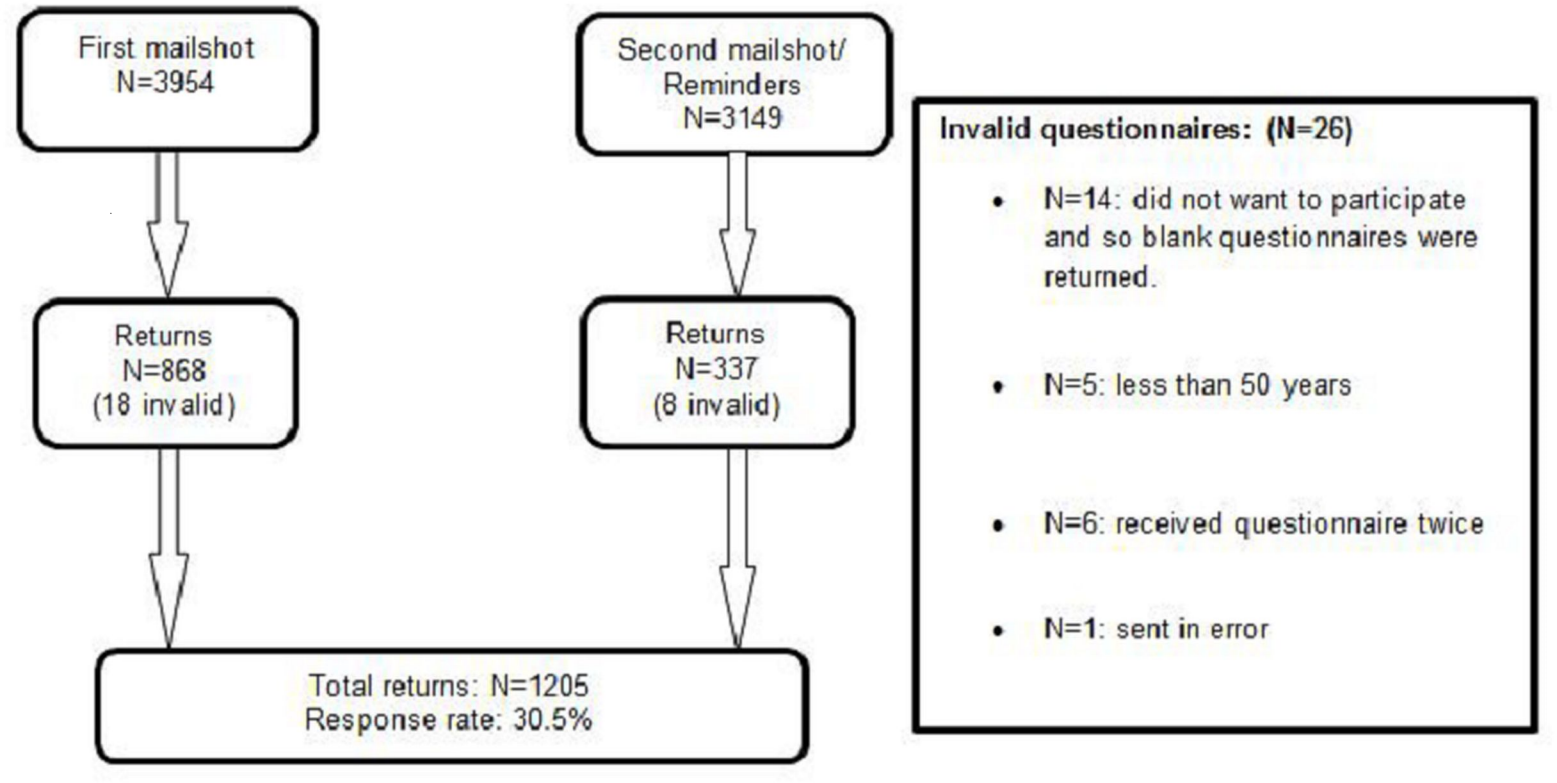
Study recruitment- Flow chart illustrating the study recruitment.

The response rate after one reminder was 30.5% (1205/3954) (Fig 1). Women and older people (60–69 years compared to 50–59 years) were more likely to respond (S1 Table). Little’s MCAR test [36] was not significant when considering the respondents’ age suggesting that data were missing completely at random (χ^2^ = 0.051; DF = 1; p = 0.821).

### Sample characteristics

The study population comprised 1205 respondents; 45.5% men and 53.1% women (Table 1), mean age 62.8 years (SD = 8.8; range = 50–98), mostly White British (97.2%). Just over half of the sample had never smoked (53.6%), with 25.8% smokers and 18.3% ex-smokers. The final sample comprised more ex-smokers than expected. Among ex-smokers, 71% had stopped smoking over 12 months prior the study, while 28% had stopped smoking within the previous 12 months. The average number of cigarettes smoked per day by 306 respondents was 12.7 (SD = 7.6, range 0–40); 26.4% were classed as heavy smokers, the proportions of moderate and light smokers were the same (36.8%). There was no evidence of an association between smoking status and age (p = 0.062), gender (p = 0.297) or ‘having a previous cardiorespiratory diagnosis’ (p = 0.473). However, there was evidence of a relationship between smoking and education (p = 0.034), accommodation (p<0.001) and cancer experience (p = 0.003).

**Table 1:** Sample characteristics, frequencies and association with smoking.

Variable		Association with smoking status						
		Never-smokers	Ex-smokers	Smokers				
	Frequency, n(%)	Frequency, n(%)	Frequency, n(%)	Frequency, n(%)	Total, n	Chi2	p value	V
**Smoking status**	n = 1196	-	-	-				
Never smokers	641 (53.6)							
Ex-smokers	219 (18.3)				-	-	-	-
Smokers	308 (25.8)							
Missing	28 (2.3)							
**Gender**	n = 1189	n = 600	n = 202	n = 269	n = 1071			
Men	541 (45.5)	264 (44.0)	101 (50.0)	127 (47.2)	492 (45.9)			
Women	631 (53.1)	336 (56.0)	101 (50.0)	142 (52.8)	579 (54.1)	2.43	0.297	0.05
Missing	17 (1.4)							
**Age**	n = 1200	n = 578	n = 195	n = 259	n = 1032			
50–59	451 (39.7)	239 (41.4)	69 (35.4)	103 (39.8)	411 (39.8)			
60–69	452 (39.8)	212 (36.7)	90 (46.2)	114 (44.0)	416 (40.3)	8.95	0.062	0.07
70+	233 (20.5)	127 (22.0)	36 (18.5)	42 (16.2)	205 (19.9)			
Missing	64 (5.3)							
**Education**	n = 1189	n = 576	n = 198	n = 258	n = 1032			
Degree	292 (24.6)	161 (28.0)	58 (29.3)	52 (20.2)	271 (26.3)	6.76	0.034	0.08
No degree	837 (70.4)	415 (72.1)	140 (70.7)	206 (79.8)	761 (73.7)			
Missing	60 (5.1)							
**Accommodation**	n = 1193	n = 598	n = 202	n = 271	n = 1071			
Own house	983 (82.4)	521 (87.1)	179 (88.6)	200 (73.8)	900 (84.0)	28.6	<0.001	0.16
Renting	194 (16.3)	77 (12.9)	23 (11.4)	71 (26.2)	171 (16.0)			
Missing	16 (1.3)							
**Cancer experience**	n = 1056	n = 582	n = 191	n = 257	n = 1030	11.3	0.003	0.1
No experience	114 (10.8)	46 (7.9)	21 (11.0)	40 (15.6)	107 (10.4)			
Have experience	941 (89.2)	536 (92.1)	170 (89.0)	217 (84.4)	923 (89.6)			
**Previous cardiorespiratory diagnosis**	n = 1057	n = 582	n = 191	n = 258	n = 1031	1.5	0.473	0.04
No	587 (55.6)	331 (56.9)	106 (55.5)	135 (52.3)	572 (55.5)			
Yes	469 (44.4)	251 (43.1)	85 (44.5)	123 (47.7)	459 (44.5)			

Key: The totals are varied because of missing data; **bold** figures indicate the statistically significant findings (p≤0.05); n = total number; v = Cramér V; Chi2 = Pearson’s correlation chi2; CI = confidence interval

### Cough

Almost a fifth of our respondents (19.2%) reported experiencing cough; of these, less than half had consulted their doctor (36.0%) (Table 2), but 60.1% of all respondents were aware that cough is a potential cancer symptom. Experience of cough was associated with smoking status (p<0.001), with experience being more common amongst smokers than never-smokers (adjusted OR = 2.56 95% CI[1.75–3.75]). Amongst the smokers (34.2%) who experienced cough, only about a quarter consulted for this (24.4%)(Table 2). Smoking status was related to consulting for cough (p = 0.011) with smokers less likely to consult than never-smokers (adjusted OR = 0.37 95%CI[0.17–0.80]) (Table 3). There was no evidence of an association between smoking and awareness that cough could be a cancer symptom (p = 0.898).

**Table 2:** Frequencies for symptoms experience, self-reported consulting and awareness of cough, breathlessness, tiredness and shoulder pain and the association with smoking status.

		Association with smoking status						
		Never-smokers	Ex-smokers	Smokers				
	Frequency, n(%)	Frequency, n(%)	Frequency, n(%)	Frequency, n(%)	Total, n (%)	Chi2	p value	V
**Cough**								
**Symptoms experience**	n = 1056	n = 563	n = 185	n = 246	n = 994	40.83	<0.001	0.2
No	814 (77.1)	475 (84.4)	158 (85.4)	162 (65.9)	795 (80.0)			
Yes	203 (19.2)	88 (15.6)	27 (14.6)	84 (34.2)	199 (20.0)			
Missing	39 (3.7)	-	-	-	-			
**Self-reported consulting**	n = 203	n = 83	n = 25	n = 82	n = 190			
Did not consult	119 (58.6)	43 (51.8)	13 (52.0)	62 (75.6)	118 (62.1)			
Consulted	73 (36.0)	40 (48.2)	12 (48.0)	20 (24.4)	72 (37.9)	11.18	0.004	0.24
Did not complete section	11 (5.4)	-	-	-	-			
**Awareness**	n = 1057	n = 583	n = 191	n = 258	n = 1032			
Not aware	422 (39.9)	229 (39.3)	74 (38.7)	111 (43.0)	414 (40.1)	1.23	0.541	0.03
Aware	635 (60.1)	354 (60.7)	117 (61.3)	147 (57.0)	618 (59.9)			
**Breathlessness**								
**Symptom experience**	n = 1056	n = 557	n = 180	n = 242	n = 979	25.26	<0.001	0.16
No	887 (84.0)	517 (92.8)	154 (85.6)	196 (81.0)	867 (88.6)			
Yes	115 (10.9)	40 (7.2)	26 (14.4)	46 (19.0)	112 (11.4)			
Missing	54 (5.1)	-	-	-	-			
**Self-reported consulting**	n = 115	n = 36	n = 24	n = 40	n = 100			
Did not consult	63 (54.8)	23 (63.9)	13 (54.2)	27 (67.5)	63 (63.0)			
Consulted	38 (33.0)	13 (36.1)	11 (45.8)	13 (32.5)	37 (37.0)	1.16	0.559	0.11
Did not complete section	14 (12.2)	-	-	-	-			
**Awareness**	n = 1057	n = 583	n = 191	n = 258	n = 1032			
Not aware	786 (74.4)	446 (76.5)	128 (67.0)	198 (76.7)	772 (74.8)	7.55	0.023	0.09
Aware	271 (25.6)	137 (23.5)	63 (33.0)	60 (23.3)	260 (25.2)			
**Tiredness**								
**Symptoms experience**	n = 1056	n = 565	n = 183	n = 243	n = 991	11.83	0.003	0.11
No	584 (55.3)	352 (62.3)	95 (51.9)	124 (51.0)	571 (57.6)			
Yes	432 (40.9)	213 (37.7)	88 (48.1)	119 (49.0)	420 (42.4)			
Missing	40 (3.8)	-	-	-	-			
**Self-reported consulting**	n = 432	n = 188	n = 80	n = 106	n = 374			
Did not consult	298 (69.0)	143 (76.1)	63 (78.8)	84 (79.3)	290 (77.5)			
Consulted	87 (20.1)	45 (23.9)	17 (21.3)	22 (20.8)	84 (22.5)	0.48	0.787	0.04
Did not complete section	47 (10.9)	-	-	-	-			
**Awareness**	n = 1057	n = 583	n = 191	n = 258	n = 1032			
Not aware	641 (60.6)	344 (59.0)	109 (57.1)	172 (66.7)	625 (60.6)	5.59	0.061	0.07
Aware	416 (39.4)	239 (41.0)	82 (42.9)	86 (33.3)	407 (39.4)			
**Shoulder pain**								
**Symptom experience**	n = 1056	n = 560	n = 185	n = 239	n = 984	4.46	0.107	0.07
No	859 (81.3)	491 (87.7)	153 (82.2)	200 (83.7)	843 (85.7)			
Yes	147 (13.9)	69 (12.3)	33 (17.8)	39 (16.3)	141 (14.3)			
Missing	50 (4.7)	-	-	-	-			
**Self-reported consulting**	n = 147	n = 63	n = 27	n = 34	n = 124			
Did not consult	76 (51.7)	42 (66.7)	16 (59.3)	15 (44.1)	73 (58.9)			
Consulted	52 (35.4)	21 (33.3)	11 (40.7)	19 (55.9)	51 (41.1)	4.64	0.098	0.19
Did not complete section	19 (12.9)	-	-	-	-			
**Awareness**	n = 1057	n = 583	n = 191	n = 258	n = 1032			
Not aware	834 (78.9)	454 (77.9)	151 (79.1)	209 (81.0)	814 (78.9)	1.06	0.589	0.03
Aware	223 (21.1)	129 (22.1)	40 (20.9)	49 (19.0)	218 (21.1)			

Key: The totals are varied because of missing data; **bold** figures indicate the statistically significant findings (p≤0.05); n = total number; % = percentage; CI = confidence interval; Adj. = adjusted; OR = odds ratio; Obs = observations; Std dev = standard deviation;v = Cramér

**Table 3:** Univariate and multivariate logistic regression findings for the association between smoking status and reporting, consulting and awareness of cough, breathlessness, tiredness and shoulder pain.

	Reported symptom			Consulting			Awareness		
	Unadjusted OR (CI: 95%)	Adjusted OR (CI: 95%)	p value	Unadjusted OR (CI: 95%)	Adjusted OR (CI: 95%)	p value	Unadjusted OR (CI: 95%)	Adjusted OR (CI: 95%)	p value
**Cough**									
**Smoking status**	n = 949	**n = 910**		n = 179	**n = 172**	** **	n = 983	n = 942	0.898
Never-smokers	1	**1**	**<0.001**	1	**1**	**0.011**	1	1	
Ex-smokers	0.90 (0.56–1.46)	**0.87 (0.52–1.43)**		1.21 (0.48–3.07)	**1.57 (0.52–4.70)**	** **	1.06 (0.76–1.50)	1.09 (0.75–1.58)	
Smokers	**2.93 (2.04–4.19)**	**2.56 (1.75–3.75)**		**0.36 (0.18–0.71)**	**0.37 (0.17–0.80)**	** **	0.92 (0.68–1.26)	1.01 (0.72–1.41)	
**Breathlessness**									
**Smoking status**	**n = 935**	**n = 896**		n = 92	n = 86		n = 983	**n = 942**	**0.035**
Never-smokers	**1**	**1**	**0.002**	1	1	0.304	1	**1**	** **
Ex-smokers	**2.08 (1.21–3.59)**	**2.01 (1.12–3.62)**		1.62 (0.55–4.78)	2.34 (0.67–8.14)		**1.58 (1.10–2.26)**	**1.65 (1.12–2.41)**	** **
Smokers	**2.94 (1.84–4.71)**	**2.39 (1.43–4.00)**		0.81 (0.30–2.15)	0.97 (0.32–2.97)		1.01 (0.71–1.44)	**1.08 (0.74–1.57)**	** **
**Tiredness**									
**Smoking status**	**n = 948**	**n = 908**	** **	n = 359	n = 345		n = 983	n = 942	0.125
Never-smokers	**1**	**1**	**0.004**	1	1	0.818	1	1	
Ex-smokers	**1.53 (1.09–2.16)**	**1.66 (1.16–2.39)**	** **	0.90 (0.48–1.71)	0.89 (0.45–1.77)		1.13 (0.81–1.58)	1.12 (0.78–1.62)	
Smokers	**1.61 (1.18–2.20)**	**1.57 (1.12–2.19)**	** **	0.83 (0.46–1.49)	0.81 (0.42–1.56)		0.73 (0.53–1.01)	0.76 (0.54–1.07)	
**Shoulder pain**									
**Smoking status**	n = 940	n = 902		n = 123	n = 119		n = 983	n = 942	
Never-smokers	1	1	0.081	1	1	0.125	1	1	0.933
Ex-smokers	1.53 (0.97–2.41)	1.69 (1.06–2.70)		1.38 (0.54–3.48)	1.37 (0.50–3.73)		0.95 (0.63–1.43)	0.97 (0.64–1.47)	
Smokers	1.37 (0.89–2.10)	1.29 (0.82–2.04)		**2.71 (1.14–6.46)**	2.68 (1.04–6.91)		0.89 (0.61–1.29)	0.93 (0.62–1.38)	

Key: **bold** figures indicate the statistically significant findings (p≤0.05); n = total number; % = percentage; CI = confidence interval; OR = odds ratio; we adjusted for age, gender, education, accommodation, cancer experience and ‘having a previous cardiorespiratory diagnosis’.

### Breathlessness

Just over a tenth of the respondents (10.9%) reported experiencing breathlessness. A third of those experiencing the symptom consulted (33.0%) but only about a quarter (25.6%) were aware that breathlessness is a potential cancer symptom (Table 2). Experience of breathlessness was associated with smoking status (p = 0.002) and was more common amongst smokers than never-smokers (adjusted OR = 2.39 95% CI[1.43–4.00]). Over a fifth of smokers were aware that breathlessness is a potential cancer symptom (23.3%). Smoking status was associated with awareness that breathlessness was a potential cancer symptom (p = 0.035) with ex-smokers more likely to be aware than never-smokers (adjusted OR = 1.65 95% CI [1.12–2.41]) (Table 3). There was no evidence of an association between smoking and consulting for breathlessness (p = 0.304).

### Tiredness

Less than half of our respondents (40.9%) reported experiencing tiredness; of these, about a fifth consulted their doctor (20.1%) (Table 2) and 39.4% of all respondents were aware that tiredness is a potential cancer symptom. Smoking status was associated with experience of tiredness (p = 0.004) with tiredness being more common amongst smokers than never-smokers (OR = 1.57 95% CI [1.12–2.19]). There was no evidence of an association between smoking and consulting for tiredness (p = 0.818) or awareness of tiredness as a potential cancer symptom (p = 0.125).

### Shoulder pain

Just over a tenth of our respondents (13.9%) reported experiencing shoulder pain. Of these, less than half had consulted (35.4%) (Table 2), but about a fifth (21.1%) of all respondents were aware that shoulder pain is a potential cancer symptom. There was no evidence of an association between smoking and experiencing shoulder pain (p = 0.081); smoking and consulting (p = 0.125) or smoking and awareness of shoulder pain as a potential cancer symptom (p = 0.933) (Table 3).

## Discussion

### Main findings

Smoking was associated with increased experience of cough, breathlessness and tiredness. Smokers were more likely than never-smokers to experience the three symptoms while ex-smokers were more likely than never-smokers to experience breathlessness and tiredness. Smoking status was associated with consulting for cough but not for the other three symptoms. Smokers were less likely to consult for cough than never-smokers. Smoking status was associated with awareness for breathlessness as a potential cancer symptom but not with the other three symptoms. Ex-smokers were more likely than never-smokers to be aware of breathlessness as a potential cancer symptom while smokers were just as aware of breathlessness as never-smokers.

### Comparison with other studies and possible explanations for our findings

Being a current smoker was associated with increased experience of cough, breathlessness and tiredness, which are among the six key symptoms associated with suspected lung cancer [33]. It was also associated with consulting for cough as confirmed by Friedemann Smith *et al* [19] Previous evidence has suggested that generally cough is not recognized as a potential cancer symptom hence the time taken to present to the doctor with cough is longer [6]. This is especially the case for smokers, who usually have a ‘smoker’s cough’ and so will normalize cough [9]. This may lead to consulting their doctor later than average [13]. Other evidence suggested that smokers were less likely than never-smokers to use preventive/cancer screening services [37]. There was no association of smoking status with consulting for the other three symptoms probably because there is generally low awareness of potential cancer symptoms [8].

Previous research suggested that breathlessness rarely occurred in isolation and that it normally occurred in combination with other symptoms associated with lung cancer [23]. This therefore highlights the importance of focusing on the multiple symptoms of lung cancer [32,33], particularly in public health media campaigns.

### Strengths and limitations

This is the first study to focus on experience of-, consulting and awareness of symptoms suggestive of lung and/or head and neck cancer in relation to smoking status. Previous research has mainly focused on a single area such as prevalence [24], awareness [7,18,21], or consulting [13,22]. There is limited evidence combining these three areas. Most evidence relating to cancer focuses on lung cancer [16,17], with less evidence on head and neck cancers [14,15].

Ours is also the first study to focus on experience of-, consulting and awareness of symptoms suggestive of lung and/or head and neck cancers among smokers over 50 years, a group at high risk of several cancers, and to sample in such a way as to obtain a reasonable return from smokers. We obtained a similar response rate to similar postal questionnaire studies [38,39]. We also achieved the proposed sample size probably because our recruitment areas had higher than average smoking prevalence than the YH and English figures [22]. Our findings for symptoms experienced and consulting were empirical unlike the awareness findings, which were not empirical findings, but reflected the respondents’ health literacy. We were therefore able to obtain estimates of symptoms experienced and consulting within our study population. Cough, breathlessness, tiredness and shoulder pain are common and are among the key symptoms for suspected lung cancer in the NICE guidelines [33] therefore these findings are clinically relevant. We did not mention ‘cancer’ in the PIS and questionnaire; we referred to the questionnaire as the ‘Symptoms Awareness Study’ to reduce the risk of biased responses from our respondents. This was done so as to reduce/prevent the respondents’ over-reporting that they consulted for a given symptom if the study had been about ‘cancer’. This way we were more likely to obtain the respondents’ true consulting behaviour.

Smoking status was self-reported, which may be a valid marker for assessing tobacco exposure [40]. However, when considering the self-reported never-smokers caution should be taken since there might be the possibility of misclassification because of stigma whereby, the ‘never-smoker’ might have lied about their smoking status. To avoid this, biochemical tests, which are more accurate, are recommended [40]. This was a postal questionnaire which suggests that people with low literacy skills may have been excluded. The study respondents were mostly White British with very limited ethnic minority involvement suggesting that these findings cannot be generalized to a multicultural community and may instead be relevant to a similar population. We do not have information on the number of ethnic minority individuals who were invited to participate in the study; therefore we do not know whether there was a non-response bias among the ethnic minority groups. The response rate was low although within the parameters of questionnaire studies [38,39], thereby suggesting the possibility of bias in the study sample. Although our criterion for recruitment was ‘no cancer diagnosis’, few respondents were diagnosed with cancer; to minimize this effect on cancer awareness, we controlled for ‘cancer experience’ in adjusted analyses. Additionally, since these symptoms are also present in other non-cancer diseases (cardiorespiratory conditions) and could influence our consulting findings, we controlled for ‘having previous cardiorespiratory diagnosis’.

### Implication

Our findings highlight the importance of targeting not only smokers but ex-smokers as they also reported experiencing breathlessness and tiredness, some of the key symptoms associated with lung cancer [33]. Our findings also suggest that cancer awareness interventions should focus on the key potential cancer symptoms rather than a singular symptom [23,32,33]. Drawing on the consulting findings, innovative interventions targeting smokers, particularly those with cough symptoms, to improve their consulting behavior should be considered. In order to understand this population’s low consulting behavior, further work on the attributions of potential cancer symptoms in older people should be considered.

## Conclusion

Our findings highlight the need to increase cancer awareness and promote consulting among smokers therefore innovative interventions improving symptom recognition and empowering smokers to seek help are required.

## Supporting information

S1 Table This table illustrates the study population representativeness. (PDF)

S1 Questionnaire Copy of the questionnaire sent to eligible study participants. (PDF)
