# Supplementary material for: Experience of, awareness of and help-seeking for potential cancer symptoms in smokers and non-smokers: A cross-sectional study
Source: PLoS One. 2017 Aug 28;12(8):e0183647. doi: 10.1371/journal.pone.0183647 (PMC5573210; doi:10.1371/journal.pone.0183647)
Supplement: S1 Table — (PDF) [file pone.0183647.s001.pdf]

S1 Table

Study representativeness						
**Variable**	**Frequency, n (%)**					
**Response**<br>Responders<br>Non-responders<br>Total	 1140 (29.6)<br>2707 (70.4)<br>3847 (100.0)					
**Variable**	**Responders**	**Non-responders**	**Total, n (%)**	**Chi^2**	**p value**	**v**
**Age**<br>50-59<br>60-69<br>70+<br>Total	 451 (39.7)<br>451 (39.7)<br>234 (20.6)<br>1136 (100..0)	 1232 (45.9)<br>863 (32.2)<br>588 (21.9)<br>2683 (100.0)	 1683 (44.1)<br>1314 (34.4)<br>822 (21.5)<br>3819 (100.0)	**20.8156**	**<0.001**	**0.07**
**Gender**<br>Men<br>Women<br>Total	 532 (46.7)<br>608 (53.3)<br>1140 (100.0)	 1399 (51.7)<br>1308 (48.3)<br>2707 (100.0)	 1931 (50.2)<br>1916 (49.8)<br>3847 (100.0)	**8.0674**	**0.005**	**-0.05**
**Response**						
	Adj OR (CI: 95%) n=3819	***p value***				
Age<br>50-59<br>60-69<br>70+	 **1**<br>**1.43 (1.22-1.67)**<br>**1.06 (0.88-1.28)**	**<0.001**				
Gender<br>Men<br>Women	 **1**<br>**1.24 (1.07-1.42)**	**0.003**				

Key: **bold** figures indicate the statistically significant findings (p≤0.05); n=total number; CI=confidence interval; Adj.=adjusted; OR=odds ratio; CI: confidence interval; we controlled for gender and age.
