# Supplementary material for: Experience of, awareness of and help-seeking for potential cancer symptoms in smokers and non-smokers: A cross-sectional study
Source: PLoS One. 2017 Aug 28;12(8):e0183647. doi: 10.1371/journal.pone.0183647 (PMC5573210; doi:10.1371/journal.pone.0183647)
Supplement: S1 Questionnaire — (PDF) [file pone.0183647.s002.pdf]

For office use only

# Symptoms Awareness Survey

Version 2-01/05/2013

**THIS PAGE HAS BEEN LEFT BLANK INTENTIONALLY.**

**Thank you for agreeing to complete this survey.**

- **It should take no longer than 20 minutes to complete. You may, however, take as long as you want.**
- **Your answers will be treated in the strictest confidence.**
- **Please return the questionnaire in the freepost envelope (no stamp required). By returning the questionnaire you are consenting to take part in the study.**

**If you would like help with this questionnaire,**
**please call: 01482 463128**

## FIRST, WE WOULD LIKE TO ASK YOU SOME QUESTIONS ABOUT YOUR HEALTH

### Do you have any illnesses or conditions that affect your daily life?

Yes ☐

No ☐

### Do you have any of the following conditions/illnesses?

**Please tick Yes for each condition/illness you have**

| | **Yes** |
|---|---|
| Arthritis | ☐ |
| Cancer | ☐ |
| Circulation problems | ☐ |
| Chest problems | ☐ |
| Cholesterol problems | ☐ |
| Depression | ☐ |
| Diabetes | ☐ |
| Heart problems | ☐ |
| High blood pressure | ☐ |
| Kidney problems | ☐ |
| Stroke | ☐ |
| Other | ☐ |

**If you have answered 'Other', please write the names of the illnesses or conditions here**

## Access to health care

Are you able to access health care services such as the doctor or practice nurse when you need to?

- All of the time ☐
- Most of the time ☐
- Sometimes ☐
- No ☐

**If NO, what stops you from accessing these services when you need to?**

**Please tick as many as apply**

- Too busy / not enough time ☐
- Difficulty getting an appointment ☐
- Financial reasons ☐
- A health condition, illness or impairment, or disability ☐
- Lack of public transport, is infrequent or unreliable ☐
- No access to a car as a driver or passenger ☐
- Fear of crime (for example mugging or robbery) ☐
- Anxiety ☐
- Lack of confidence in doctors or nurses ☐
- Other reasons ☐

If you have ticked 'Other reasons', please write them down here

**The next section asks you about symptoms or changes you have noticed in your health that you have had in the last 3 months.**

- **Please read the 17 symptoms down the left hand side and if you haven't had it, tick 'NO' and move down the page to the next symptom.**
- **If you tick 'YES', then please answer the other questions about the symptoms which are across the top of the page.**

**PLEASE TURN OVER THE PAGE**

THESE QUESTIONS ARE ABOUT YOUR EXPERIENCE OF SYMPTOMS IN THE **LAST 3 MONTHS**.

***Please read the 17 symptoms down the left hand side and if you haven't had it, tick 'NO' and move down the page to the next symptom.***
***If you tick 'YES', then please answer the other questions about the symptoms which are across the top of the page.***

| **In the last 3 months or so, have you had the following….?** *Please give an answer for each symptom* | **Were you worried about it?** | **How much did it interfere with your life?** | **What did you think caused it? (Please write)** | **Did you contact your doctor about it?** | **If you did contact your doctor, what was the outcome?** |
|---|---|---|---|---|---|
| **1. Cough that didn't go away**<br>☐ **NO** ☐ **YES** → | ☐ Not at all<br>☐ A little bit<br>☐ Moderately<br>☐ Quite a bit<br>☐ Extremely | ☐ Not at all<br>☐ A little bit<br>☐ Moderately<br>☐ Quite a bit<br>☐ Extremely | ____________________<br>____________________ | ☐ No<br>☐Yes, within 1-2 weeks<br>☐Yes, within 1 month<br>☐Yes, within 6 weeks<br>☐Yes, within 3 months | ☐ further tests<br>☐ diagnosis<br>☐ treatment<br>☐ told to come back |
| **2. Unexplained shortness of breath**<br>☐ **NO** ☐ **YES** → | ☐ Not at all<br>☐ A little bit<br>☐ Moderately<br>☐ Quite a bit<br>☐ Extremely | ☐ Not at all<br>☐ A little bit<br>☐ Moderately<br>☐ Quite a bit<br>☐ Extremely | ____________________<br>____________________ | ☐ No<br>☐Yes, within 1-2 weeks<br>☐Yes, within 1 month<br>☐Yes, within 6 weeks<br>☐Yes, within 3 months | ☐ further tests<br>☐ diagnosis<br>☐ treatment<br>☐ told to come back |
| **3. Unexplained weight loss**<br>☐ **NO** ☐ **YES** → | ☐ Not at all<br>☐ A little bit<br>☐ Moderately<br>☐ Quite a bit<br>☐ Extremely | ☐ Not at all<br>☐ A little bit<br>☐ Moderately<br>☐ Quite a bit<br>☐ Extremely | ____________________<br>____________________ | ☐ No<br>☐Yes, within 1-2 weeks<br>☐Yes, within 1 month<br>☐Yes, within 6 weeks<br>☐Yes, within 3 months | ☐ further tests<br>☐ diagnosis<br>☐ treatment<br>☐ told to come back |
| **4. Chest infection that didn't go away**<br>☐ **NO** ☐ **YES** → | ☐ Not at all<br>☐ A little bit<br>☐ Moderately<br>☐ Quite a bit<br>☐ Extremely | ☐ Not at all<br>☐ A little bit<br>☐ Moderately<br>☐ Quite a bit<br>☐ Extremely | ____________________<br>____________________ | ☐ No<br>☐Yes, within 1-2 weeks<br>☐Yes, within 1 month<br>☐Yes, within 6 weeks<br>☐Yes, within 3 months | ☐ further tests<br>☐ diagnosis<br>☐ treatment<br>☐ told to come back |

| In the last 3 months or so, have you had the following….? *Please give an answer for each symptom* | Were you worried about it? | How much did it interfere with your life? | What did you think caused it? (Please write) | Did you contact your doctor about it? | If you did contact your doctor, what was the outcome? |
|---|---|---|---|---|---|
| **5. Tiredness or lack of energy**<br>☐ **NO** ☐ **YES** → | ☐ Not at all<br>☐ A little bit<br>☐ Moderately<br>☐ Quite a bit<br>☐ Extremely | ☐ Not at all<br>☐ A little bit<br>☐ Moderately<br>☐ Quite a bit<br>☐ Extremely | ____________________<br>____________________ | ☐ No<br>☐Yes, within 1-2 weeks<br>☐Yes, within 1 month<br>☐Yes, within 6 weeks<br>☐Yes, within 3 months | ☐ further tests<br>☐ diagnosis<br>☐ treatment<br>☐ told to come back |
| **6. Chest pain that didn't go away**<br>☐ **NO** ☐ **YES** → | ☐ Not at all<br>☐ A little bit<br>☐ Moderately<br>☐ Quite a bit<br>☐ Extremely | ☐ Not at all<br>☐ A little bit<br>☐ Moderately<br>☐ Quite a bit<br>☐ Extremely | ____________________<br>____________________ | ☐ No<br>☐Yes, within 1-2 weeks<br>☐Yes, within 1 month<br>☐Yes, within 6 weeks<br>☐Yes, within 3 months | ☐ further tests<br>☐ diagnosis<br>☐ treatment<br>☐ told to come back |
| **7. Shoulder pain that didn't go away**<br>☐ **NO** ☐ **YES** → | ☐ Not at all<br>☐ A little bit<br>☐ Moderately<br>☐ Quite a bit<br>☐ Extremely | ☐ Not at all<br>☐ A little bit<br>☐ Moderately<br>☐ Quite a bit<br>☐ Extremely | ____________________<br>____________________ | ☐ No<br>☐Yes, within 1-2 weeks<br>☐Yes, within 1 month<br>☐Yes, within 6 weeks<br>☐Yes, within 3 months | ☐ further tests<br>☐ diagnosis<br>☐ treatment<br>☐ told to come back |
| **8. Coughing up blood**<br>☐ **NO** ☐ **YES** → | ☐ Not at all<br>☐ A little bit<br>☐ Moderately<br>☐ Quite a bit<br>☐ Extremely | ☐ Not at all<br>☐ A little bit<br>☐ Moderately<br>☐ Quite a bit<br>☐ Extremely | ____________________<br>____________________ | ☐ No<br>☐Yes, within 1-2 weeks<br>☐Yes, within 1 month<br>☐Yes, within 6 weeks<br>☐Yes, within 3 months | ☐ further tests<br>☐ diagnosis<br>☐ treatment<br>☐ told to come back |

| In the last 3 months or so, have you had the following….? *Please give an answer for each symptom* | Were you worried about it? | How much did it interfere with your life? | What did <u>you</u> think caused it? (Please write) | Did you contact your doctor about it? | If you did contact your doctor, what was the outcome? |
|---|---|---|---|---|---|
| **9. Painful cough**<br>☐ **NO** ☐ **YES** → | ☐ Not at all<br>☐ A little bit<br>☐ Moderately<br>☐ Quite a bit<br>☐ Extremely | ☐ Not at all<br>☐ A little bit<br>☐ Moderately<br>☐ Quite a bit<br>☐ Extremely | ____________________<br>____________________ | ☐ No<br>☐Yes, within 1-2 weeks<br>☐Yes, within 1 month<br>☐Yes, within 6 weeks<br>☐Yes, within 3 months | ☐ further tests<br>☐ diagnosis<br>☐ treatment<br>☐ told to come back |
| **10. Loss of appetite**<br>☐ **NO** ☐ **YES** → | ☐ Not at all<br>☐ A little bit<br>☐ Moderately<br>☐ Quite a bit<br>☐ Extremely | ☐ Not at all<br>☐ A little bit<br>☐ Moderately<br>☐ Quite a bit<br>☐ Extremely | ____________________<br>____________________ | ☐ No<br>☐Yes, within 1-2 weeks<br>☐Yes, within 1 month<br>☐Yes, within 6 weeks<br>☐Yes, within 3 months | ☐ further tests<br>☐ diagnosis<br>☐ treatment<br>☐ told to come back |
| **11. Difficulty in swallowing**<br>☐ **NO** ☐ **YES** → | ☐ Not at all<br>☐ A little bit<br>☐ Moderately<br>☐ Quite a bit<br>☐ Extremely | ☐ Not at all<br>☐ A little bit<br>☐ Moderately<br>☐ Quite a bit<br>☐ Extremely | ____________________<br>____________________ | ☐ No<br>☐Yes, within 1-2 weeks<br>☐Yes, within 1 month<br>☐Yes, within 6 weeks<br>☐Yes, within 3 months | ☐ further tests<br>☐ diagnosis<br>☐ treatment<br>☐ told to come back |
| **12. Change in an existing cough**<br>☐ **NO** ☐ **YES** → | ☐ Not at all<br>☐ A little bit<br>☐ Moderately<br>☐ Quite a bit<br>☐ Extremely | ☐ Not at all<br>☐ A little bit<br>☐ Moderately<br>☐ Quite a bit<br>☐ Extremely | ____________________<br>____________________ | ☐ No<br>☐Yes, within 1-2 weeks<br>☐Yes, within 1 month<br>☐Yes, within 6 weeks<br>☐Yes, within 3 months | ☐ further tests<br>☐ diagnosis<br>☐ treatment<br>☐ told to come back |
| **13. Mouth ulcer that didn't heal**<br>☐ **NO** ☐ **YES** → | ☐ Not at all<br>☐ A little bit<br>☐ Moderately<br>☐ Quite a bit<br>☐ Extremely | ☐ Not at all<br>☐ A little bit<br>☐ Moderately<br>☐ Quite a bit<br>☐ Extremely | ____________________<br>____________________ | ☐ No<br>☐Yes, within 1-2 weeks<br>☐Yes, within 1 month<br>☐Yes, within 6 weeks<br>☐Yes, within 3 months | ☐ further tests<br>☐ diagnosis<br>☐ treatment<br>☐ told to come back |

| In the last 3 months or so, have you had the following….?<br>*Please give an answer for each symptom* | Were you worried about it? | How much did it interfere with your life? | What did <u>you</u> think caused it?<br>(Please write) | Did you contact your doctor about it? | If you did contact your doctor, what was the outcome? |
|---|---|---|---|---|---|
| **14. Numbness of lip or tongue**<br>☐ **NO** ☐ **YES** → | ☐ Not at all<br>☐ A little bit<br>☐ Moderately<br>☐ Quite a bit<br>☐ Extremely | ☐ Not at all<br>☐ A little bit<br>☐ Moderately<br>☐ Quite a bit<br>☐ Extremely | ____________________<br>____________________ | ☐ No<br>☐Yes, within 1-2 weeks<br>☐Yes, within 1 month<br>☐Yes, within 6 weeks<br>☐Yes, within 3 months | ☐ further tests<br>☐ diagnosis<br>☐ treatment<br>☐ told to come back |
| **15. Cold sore or cut on lip that didn't go away**<br>☐ **NO** ☐ **YES** → | ☐ Not at all<br>☐ A little bit<br>☐ Moderately<br>☐ Quite a bit<br>☐ Extremely | ☐ Not at all<br>☐ A little bit<br>☐ Moderately<br>☐ Quite a bit<br>☐ Extremely | ____________________<br>____________________ | ☐ No<br>☐Yes, within 1-2 weeks<br>☐Yes, within 1 month<br>☐Yes, within 6 weeks<br>☐Yes, within 3 months | ☐ further tests<br>☐ diagnosis<br>☐ treatment<br>☐ told to come back |
| **16. Hoarse voice that lasted for more than 3 weeks**<br>☐ **NO** ☐ **YES** → | ☐ Not at all<br>☐ A little bit<br>☐ Moderately<br>☐ Quite a bit<br>☐ Extremely | ☐ Not at all<br>☐ A little bit<br>☐ Moderately<br>☐ Quite a bit<br>☐ Extremely | ____________________<br>____________________ | ☐ No<br>☐Yes, within 1-2 weeks<br>☐Yes, within 1 month<br>☐Yes, within 6 weeks<br>☐Yes, within 3 months | ☐ further tests<br>☐ diagnosis<br>☐ treatment<br>☐ told to come back |
| **17. Lump in your neck**<br>☐ **NO** ☐ **YES** → | ☐ Not at all<br>☐ A little bit<br>☐ Moderately<br>☐ Quite a bit<br>☐ Extremely | ☐ Not at all<br>☐ A little bit<br>☐ Moderately<br>☐ Quite a bit<br>☐ Extremely | ____________________<br>____________________ | ☐ No<br>☐Yes, within 1-2 weeks<br>☐Yes, within 1 month<br>☐Yes, within 6 weeks<br>☐Yes, within 3 months | ☐ further tests<br>☐ diagnosis<br>☐ treatment<br>☐ told to come back |

## THE NEXT QUESTION IS ABOUT YOUR UNDERSTANDING OF SOME ILLNESSES

**Here is a list of symptoms and illnesses. For each symptom, please tick which illness you think the symptom might be related to. You can tick as many illnesses that you think are related to each symptom.**

| | Heart disease | Flu | Cancer | Asthma | Don't know |
|---|---|---|---|---|---|
| Sore throat | ☐ | ☐ | ☐ | ☐ | ☐ |
| Cough that does not go away | ☐ | ☐ | ☐ | ☐ | ☐ |
| Chest pain | ☐ | ☐ | ☐ | ☐ | ☐ |
| Unexplained pain that does not go away | ☐ | ☐ | ☐ | ☐ | ☐ |
| Difficulty swallowing | ☐ | ☐ | ☐ | ☐ | ☐ |
| Shortness of breath | ☐ | ☐ | ☐ | ☐ | ☐ |
| A mouth ulcer that does not heal | ☐ | ☐ | ☐ | ☐ | ☐ |
| Feeling tired or having low energy | ☐ | ☐ | ☐ | ☐ | ☐ |
| Unexplained weight loss | ☐ | ☐ | ☐ | ☐ | ☐ |
| Feeling your heart pound or race | ☐ | ☐ | ☐ | ☐ | ☐ |
| Shoulder pain that does not go away | ☐ | ☐ | ☐ | ☐ | ☐ |
| Coughing up blood | ☐ | ☐ | ☐ | ☐ | ☐ |
| Hoarse voice for more than 3 weeks | ☐ | ☐ | ☐ | ☐ | ☐ |
| A cold sore or cut on lip that does not go away | ☐ | ☐ | ☐ | ☐ | ☐ |
| Lump in your neck | ☐ | ☐ | ☐ | ☐ | ☐ |

| NOW WE WANT TO ASK YOU ABOUT GOING TO SEE THE DOCTOR | |
|---|---|
| **How often do you visit your doctor (GP)?**<br>**Please tick one box only** | |
| Less often than once a year | ☐ |
| About once a year | ☐ |
| About every six months | ☐ |
| About every three months | ☐ |
| At least once a month | ☐ |
| Most weeks | ☐ |
| **When you experience a new health problem that worries you, what is the first thing that you are likely to do?**<br>**Please tick one box only.** | |
| Do nothing | ☐ |
| Talk to friends or family | ☐ |
| Wait to see if it will go away | ☐ |
| Go to see your doctor | ☐ |
| Go onto the internet to try and find out what it might be | ☐ |
| Go to the pharmacy to buy some medicine | ☐ |
| Wait a couple of weeks, and if the symptoms persist, then go to see the doctor | ☐ |

# FINALLY, A FEW QUESTIONS ABOUT YOU

**What is your age and gender?**

**Age:** years | Male ☐ | Female ☐

**What is your relationship status?** **Please tick the box that best describes you**

| | |
|---|---|
| I am single | ☐ |
| I live with my husband/wife/partner | ☐ |
| I am widowed | ☐ |
| I am separated/divorced | ☐ |
| I have a partner who does not live with me | ☐ |

**What is the highest level of education qualification you have obtained?**
**Please tick one box only**

| | | | |
|---|---|---|---|
| Degree or higher degree | ☐ | O Level or GCSE (Grade D - G) | ☐ |
| A Levels or Highers | ☐ | No formal qualifications | ☐ |
| HNC/HND | ☐ | Still studying | ☐ |
| ONC/BTEC | ☐ | Other............................ | ☐ |
| O Level or GCSE equivalent (Grade A-C) | ☐ | | |

**Please tick which best describes your living arrangements:**

| | |
|---|---|
| Own house (outright) | ☐ |
| Own house (mortgage) | ☐ |
| Rent from Local Authority/ Housing Association | ☐ |
| Rent privately | ☐ |
| Other (e.g. living with family/ friends) | ☐ |

**Do you live alone?** Yes ☐ No ☐ **If No, how many people live in your house?** ☐

**Does your household own a car or van?** **Please tick one box only**

No ☐ Yes, one ☐ Yes, more than one ☐

**Have you, your family or close friends had cancer?** **Please tick one box for each person in the list**

| | Yes | No | Don't know |
|---|---|---|---|
| You | ☐ | ☐ | ☐ |
| Partner/husband/wife (if applicable) | ☐ | ☐ | ☐ |
| Close family member | ☐ | ☐ | ☐ |
| Other family member | ☐ | ☐ | ☐ |
| Close friend | ☐ | ☐ | ☐ |
| Other friend | ☐ | ☐ | ☐ |

| Do you have someone that you can talk to if you are having problems? | Yes ☐ No ☐ |
|---|---|

| **Which of these best describes you?** Please tick one box only | |
|---|---|
| I have never smoked | ☐ |
| I used to smoke regularly but I am now an ex-smoker | ☐ |
| • If you answered ex-smoker, did you give up in the last 12 months? | Yes ☐ No ☐ |
| I am a current smoker | ☐ |
| **If you answered current smoker, how much do you smoke?** Please tick one box only | |
| If you smoke cigarettes - how many do you smoke on an average day? | Number ☐☐ |
| If you smoke tobacco, how many oz do you smoke in an average week? | ☐ oz |

**Which of these best describes your ethnic group?** Please tick one box only

| White | Mixed | Asian or Asian British | Black or Black British | Chinese other |
|---|---|---|---|---|
| White British ☐ | White and Black Caribbean ☐ | Indian ☐ | Black Caribbean ☐ | Chinese ☐ |
| White Irish ☐ | White and Black African ☐ | Pakistani ☐ | Black African ☐ | Other..... ☐ |
| White, other European ☐ | White and Asian ☐ | Bangladeshi ☐ | Any other Black background ☐ | |
| Any other White background ☐ | Any other mixed background ☐ | Any other Asian background ☐ | | |

| **If you haven't lived in the UK all your life, how many years have you been living in the UK?** | ☐☐ |
|---|---|

**THANK YOU VERY MUCH FOR COMPLETING THIS QUESTIONNAIRE**

IMPORTANT:

IF YOU ARE WORRIED ABOUT ANY SYMPTOMS <u>OR</u> IF YOU ARE EXPERIENCING PERSISTENT SYMPTOMS YOU SHOULD GO TO YOUR GP FOR ADVICE

**THIS PAGE HAS BEEN LEFT BLANK INTENTIONALLY.**

# FURTHER INFORMATION

**We would like to contact some people who take part in this survey to tell us more about their health and experiences in an interview. Would you be prepared to be contacted again?**

**Yes ☐ No ☐**

If yes, please provide your details below (this information will be treated in the strictest confidence):

Name ____________________________________________

Address __________________________________________

_________________________________________________

_________________________________________________

_________________________________________________

Email ___________________________________________

Telephone ________________________________________

**If you would like to receive a summary of the results from this survey when it is completed, please fill in your contact details below.**

***Information provided here <u>will not</u> be used to contact you about taking part in any other research.***

Name ____________________________________________

Address __________________________________________

_________________________________________________

_________________________________________________

_________________________________________________

Email ___________________________________________

For office use only ☐☐☐☐☐
